# Effective natural inhibitors targeting poly ADP-ribose polymerase by computational study

**DOI:** 10.18632/aging.103986

**Published:** 2021-01-23

**Authors:** Sheng Zhong, Bo Wu, Wenzhuo Yang, Junliang Ge, Xiangheng Zhang, Zhenghe Chen, Hao Duan, Zhenqiang He, Yibing Liu, Hongyu Wang, Yuting Jiang, Zhiyun Zhang, Xinhui Wang, Weihang Li, Naimeng Liu, Xiaoyu Guo, Yonggao Mou

**Affiliations:** 1 Neurosurgery and Neuro-Oncology Department, Sun Yat-Sen University Cancer Center, State Key Laboratory of Oncology in South China, Collaborative Innovation Center for Cancer Medicine, Guangzhou, China; 2 Clinical College, Jilin University, Changchun, China; 3 Department of Orthopaedics, The First Hospital of Jilin University, Changchun, China; 4 Department of Oncology, The First Bethune Hospital of Jilin University, Changchun, China; 5 Department of Orthopaedics, Xijing Hospital, The Fourth Military Medical University, Xi’an, China

**Keywords:** glioblastoma, PARP, natural products, drug treatment, virtual screening

## Abstract

Object: This study was designed to screen ideal lead compounds and drug candidates with an inhibitory effect on PARP from the drug library (ZINC database).

Results: Two effective natural compounds ZINC000003938684 and ZINC000014811844 were found to bind to PARP in the ZINC database, showing a higher binding affinity. Also, they were predicted to have lower rodent carcinogenicity, Ames mutagenicity, developmental toxicity potential, and high tolerance to cytochrome P4502D6. Molecular dynamics simulation showed that ZINC000003938684 and ZINC000014811844 had a more favorable potential energies with PARP, which could exist stably in natural circumstances.

Conclusion: This study suggested that ZINC000003938684 and ZINC000014811844 were ideal potential inhibitors of PARP targeting. These compounds were safe drug candidates and had important implications for the design and improvement of CMET target drugs.

Methods: A battery of computer-aided virtual techniques were used to identify potential inhibitors of PARP. LibDock is used for structure-based screening followed by ADME (absorption distribution, metabolic excretion) and toxicity prediction. Molecular docking was performed to demonstrate the binding affinity mechanism between the ligand and PARP. Molecular dynamics simulations were used to evaluate the stability of ligand-receptor complexes.

## INTRODUCTION

Glioma is one of the most common primary malignant tumors of the central nervous system in adults [1]. Glioblastoma (GBM) accounts for more than 50% of the incidence of glioma [2], which is the most invasive subtype. The median survival time of the patients is about 18 months [3]. The current standard treatment for GBM includes surgery combined with radiotherapy and chemotherapy. However, the overall prognosis is still very poor, the median survival time of patients is about 18 months, only about 30% of patients achieve 2-year survival rate, and less than 10% of patients survive more than 3 years [4].

PARP (poly ADP-ribose polymerase) is a kind of nuclear enzyme that catalyzes the ribosylation of ADP [5]. The PARP family consists of 18 members, all of which contain highly conserved PARP catalytic sequences [6]. Among the many enzymes involved in DNA repair, PARP plays an important role. Among them, PARP-1 and PARP-2 are the main targets for the clinical use of PARP inhibitors. PARP is a sensor for DNA damage, which can quickly identify and bind to the damaged site of DNA. Through the formation of poly (ADP—ribose) polymerase (also known as "PAR"), on the one hand, it can prevent the recombination of nearby DNA molecules with damaged DNA; on the other hand, it can reduce the use of damaged DNA by exonucleases and attract DNA repair proteins to repair the damaged site [7].

In a word, antineoplastic drugs such as alkylated camptothecin kill tumors by producing a large amount of DNA damage [8], but PARP can repair tumor cells damaged by alkylating agents, which is an important reason for the failure of tumor chemotherapy. Therefore, the selection of effective PARP kinase inhibitors plays an important role in drug development and cancer treatment. At present, the third generation of PARP inhibitors has been developed [9]. Olaparib is the first FDA-approved PARP1/2 inhibitor for the treatment of ovarian cancer patients with BRCA gene deficiency [10]. The drug used in the treatment of breast, stomach, scales, prostate and other malignant solid tumors has also entered the clinical trial stage [11]. Olaparib can bind to the catalytic domain of PARP1 and inhibit its PAR alkylation activity. Therefore, Olaparib can inhibit PARP1-mediated repair of single-strand DNA damage, resulting in the transition from single-strand breaks to double-strand breaks during DNA replication [12]. Therefore, PARP inhibitors are potential adjuvants for these anti-tumor treatments. This study aimed to screen natural compounds from natural drugs that are more effective than Olaparib in treating cancer.

Natural products, as lead compounds, can be transformed into new drugs through appropriate structural modification, which is an important source of new drug research in the pharmaceutical industry [13]. In recent years, several targeted drugs have been reported to inhibit PARP [5, 10, 14]. In this study, a series of structural biological and chemical methods (including virtual screening, molecular docking, etc.) were used to screen and identify lead compounds with potential regulatory functions for PARP. Our study also predicted the absorption, distribution, metabolism, excretion and toxicity of these compounds. This study provides a list of drug candidates and their pharmacological properties, providing the research object for the development of PARP inhibitors.

## RESULTS

### Virtual screening of natural products database against PARP

The ligand-binding pocket played an important part in the regulatory sites of PARP. Therefore, this pocket region was chosen as the reference site. A total of 17931 ligands were screened from the ZINC15 database, which was marked as for-sale, biogenic and named. Select the chemical structure of PARP as the receptor to contrast the pharmacologic properties between it and other compounds. Among these, 3461 compounds had higher scores than Olaparib and the compounds which scored in the top 20 were listed in Table 1.

**Table 1:** Top 20 ranked compounds with LibDock scores.

**Number**	**Compounds**	**Libdock score**
1	ZINC000049784088	178.603
2	ZINC000003995616	177.018
3	ZINC000028968101	174.098
4	ZINC000002033588	173.054
5	ZINC000008214470	172.019
6	ZINC000049872065	168.312
7	ZINC000021992902	168.289
8	ZINC000042851784	167.67
9	ZINC000003938684	166.742
10	ZINC000001577210	165.461
11	ZINC000004098458	164.436
12	ZINC000004098657	162.95
13	ZINC000030726940	162.74
14	ZINC000002572533	162.423
15	ZINC000003979028	161.622
16	ZINC000014811844	160.521
17	ZINC000013451339	160.019
18	ZINC000004098643	159.339
19	ZINC000031298217	158.987
20	ZINC000044361207	158.911

### ADME and toxicity prediction

ADME module of Discovery Studio 4.5 was used to predict the Pharmacologic properties of the whole selected ligands and Olaparib first, including aqueous solubility level, blood-brain barrier level, CYP2D6 binding, human intestinal absorption level, hepatotoxicity and plasma protein binding properties (Table 2). According to aqueous solubility prediction (defined in water at 25 C), most of the compounds could dissolve in water. As to blood-brain barrier, all the compounds had undefined levels except ZINC000001577210 and Olaparib Three quarters of the compounds were predicted to be non-inhibitors CYP2D6, which had a great influence on drug metabolism. As for hepatoxicity, 12 compounds were found to be nontoxic, which was similar to Olaparib. For human intestinal absorption, only ZINC000001577210 and Olaparib were predicted to have good absorption. Plasma protein binding properties showed 8 compounds had weak absorption.

**Table 2:** Adsorption, distribution, metabolism, and excretion properties of compounds.

**Number**	**Compounds**	**Solubility Level**	**BBB Level**	**CYP2D6**	**Hepatotoxicity**	**Absorption Level**	**PPB Level**
1	ZINC000049784088	4	4	1	1	3	1
2	ZINC000003995616	1	4	1	1	2	0
3	ZINC000028968101	1	4	0	0	3	0
4	ZINC000002033588	2	4	0	1	3	1
5	ZINC000008214470	1	4	1	0	3	1
6	ZINC000049872065	3	4	1	1	2	1
7	ZINC000021992902	3	4	1	1	1	1
8	ZINC000042851784	0	4	1	0	2	1
9	ZINC000003938684	3	4	1	0	3	0
10	ZINC000001577210	2	1	1	1	0	0
11	ZINC000004098458	3	4	1	1	3	1
12	ZINC000004098657	3	4	1	0	3	1
13	ZINC000030726940	0	4	1	0	3	1
14	ZINC000002572533	2	4	0	1	3	1
15	ZINC000003979028	2	4	1	0	3	1
16	ZINC000014811844	3	4	1	0	2	1
17	ZINC000013451339	1	4	0	0	2	0
18	ZINC000004098643	2	4	1	1	2	0
19	ZINC000031298217	2	4	1	0	2	1
20	ZINC000044361207	0	4	0	0	3	1
21	olaparib	3	3	1	0	0	0

BBB, blood-brain barrier; CYP2D6, cytochrome P-450 2D6; PPB, plasma protein binding.

Aqueous-solubility level: 0, extremely low; 1, very low, but possible; 2, low; 3, good.

BBB level: 0, very high penetrant; 1, high; 2, medium; 3, low; 4, undefined.

CYP2D6 level: 0, noninhibitor; 0

1, inhibitor.

Hepatotoxicity: 0, nontoxic; 1, toxic.

Human-intestinal absorption level: 0, good; 1, moderate; 2, poor; 3, very poor.

PPB: 0, absorbent weak; 1, absorbent strong.

**Figure 1:**
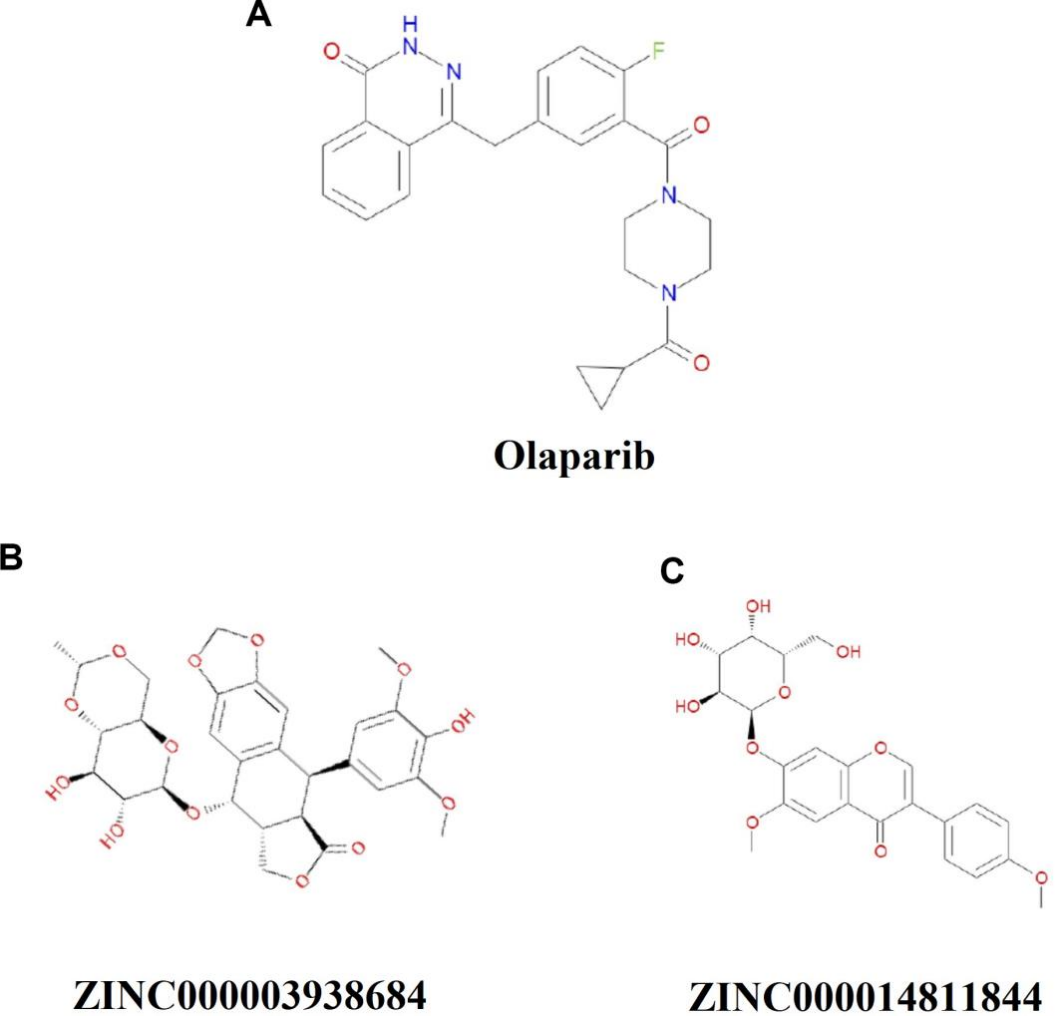
**Chemical structure formula of Olaparib and novel compounds selected from virtual screening.**

Safety ought to be greatly considered during the study. To ensure the safety of these 20 compounds, various types of toxicity indexes of the compounds and Olaparib, such as developmental toxicity potential properties, rodent carcinogenicity (based on the U.S. National Toxicology Program dataset), as well as Ames mutagenicity were predicted by using a computational method in the TOPKAT module (Table 3). Consequence illustrated 10 compounds were found to be non-mutagenic, and 3 compounds were found with no developmental toxicity potential. It is predicted that Olaparib had higher rodent carcinogenicity both in mouse and rat. In consideration of all the above results, ZINC000003938684 and ZINC000014811844 were determined to be the perfect lead compounds with non-CYP2D6 inhibitors, thus without hepatotoxicity, together with less Ames mutagenicity, developmental toxicity potential and rodent carcinogenicity in comparison with other compounds. To sum up, ZINC000003938684 and ZINC000014811844 were regarded as safe drugs and chosen for the following study (Figure 1).

**Table 3:** Toxicities of compounds.

**Number**	**Compounds**	**Mouse NTP**		**Rat NTP**		**Ames**	**DTP**
		**Female**	**Male**	**Female**	**Male**		
1	ZINC000049784088	0.995	0	0	0.008	1	1
2	ZINC000003995616	0.003	0	0.003	0	0	0.937
3	ZINC000028968101	1	0.021	0.06	0.997	1	1
4	ZINC000002033588	0	1	1	0.05	0.265	1
5	ZINC000008214470	0.939	1	1	0.999	0	1
6	ZINC000049872065	0.353	0	0.752	0.006	0	0
7	ZINC000021992902	0.198	0	0.033	0.251	0	0
8	ZINC000042851784	0	0	0	1	0.089	0.997
9	ZINC000003938684	0.025	0.953	1	0.026	0	1
10	ZINC000001577210	0	0.173	0	0.952	0	0.04
11	ZINC000004098458	0.005	0	0.988	0.003	0	1
12	ZINC000004098657	0	0.96	1	0.012	1	1
13	ZINC000030726940	0	0	0.053	1	0.983	0.411
14	ZINC000002572533	0	1	1	0.051	0.238	1
15	ZINC000003979028	1	1	0	1	0.992	0.996
16	ZINC000014811844	0.656	1	1	1	0.002	1
17	ZINC000013451339	0	0.943	0	0.038	0	1
18	ZINC000004098643	0.997	0	1	0	0	0.995
19	ZINC000031298217	0.979	1	0	0.984	0	1
20	ZINC000044361207	0	1	1	0	1	0.46
21	Olaparib	0.998	0.996	1	1	0	1

NTP, U.S.National Toxicology Program; DTP, developmental toxicity potential.

NTP<0.3 (noncarcinogen); >0.8 (carcinogen).

Ames<0.3 (nonmutagen) ;>0.8 (mutagen).

DTP<0.3 (nontoxic); >0.8 (toxic).

### Analysis of ligand binding

**Figure 2:**
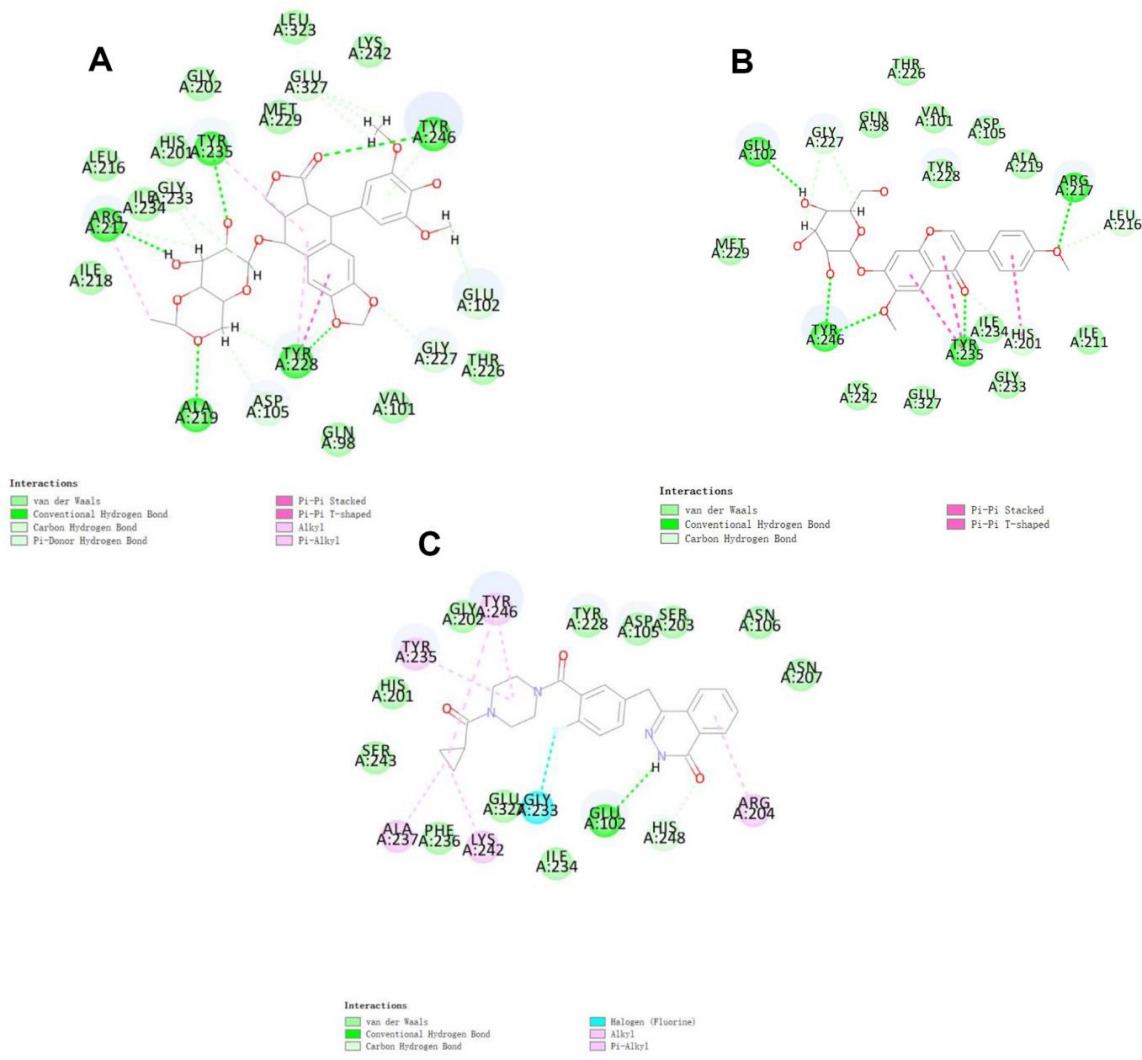
Schematic of intermolecular interaction of the predicted binding modes of (**A**) ZINC000003938684 with PARP, (**B**) ZINC000014811844 with PARP, and (**C**) Olaparib with PARP.

**Figure 3:**
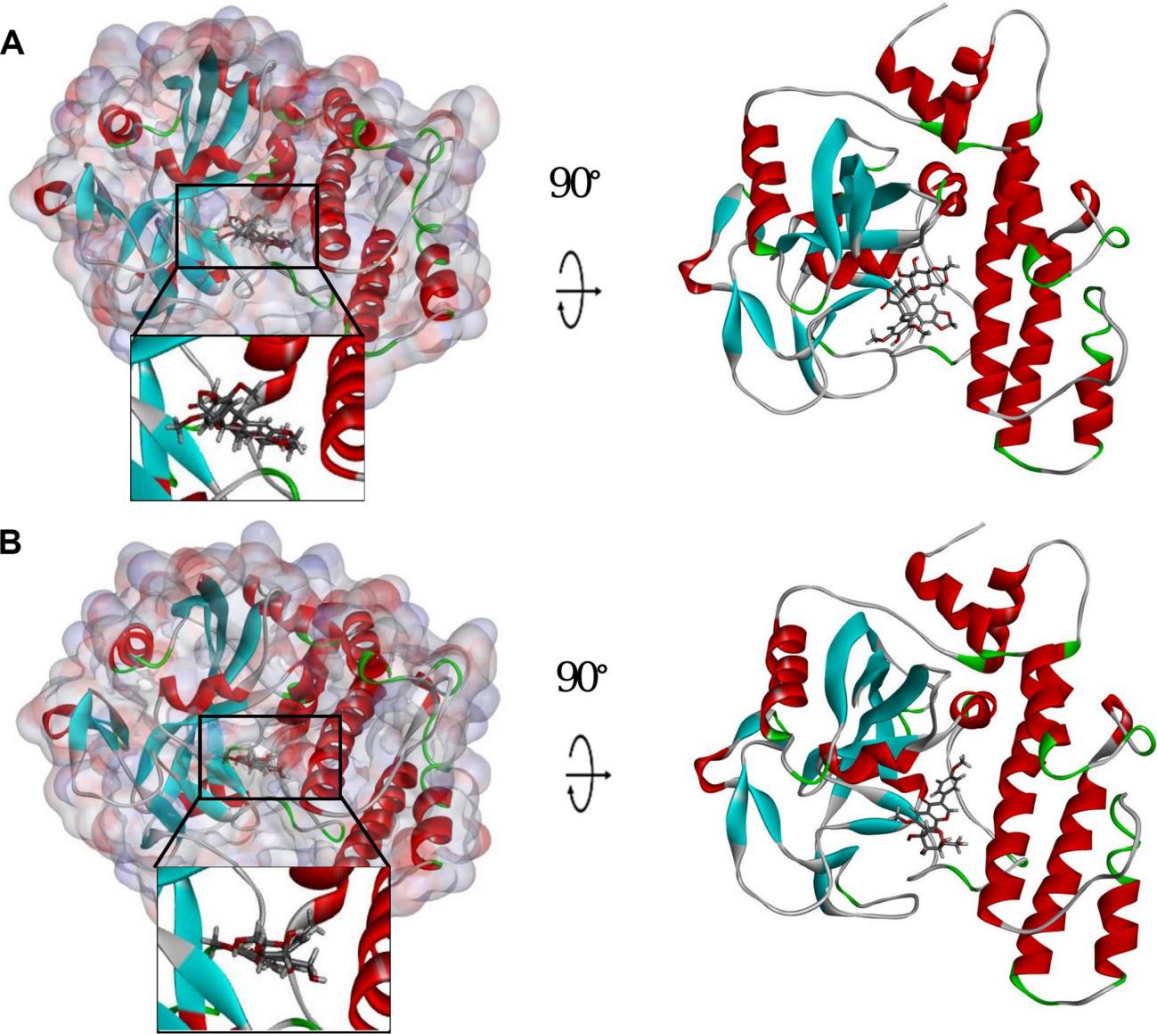
**Schematic drawing of interactions between ligands and PARP.** The surface of binding area was added. Blue represents positive charge; red represents negative charge; and ligands are shown in sticks, with the structure around the ligand-receptor junction shown in thinner sticks. (**A**) ZINC000003938684-PARP complex. (**B**) ZINC000014811844-PARP complex.

To study ligand blinding mechanisms of these compounds with 2RCW. ZINC000003938684 and ZINC000014811844 were docked into the molecule structure of 2RCW by CDOCKER module, and CDOCKER potential energy was calculated and displayed as shown in Table 4. The CDOCKER potential energy of ZINC000003938684 and ZINC000014811844 were significantly lower than the reference ligand Olaparib in the Table 4, which illustrated that 2RCW may have a higher binding affinity with ZINC000003938684 and ZINC000014811844 than Olaparib. Hydrogen bonds and π-related interactions were also performed through a structural computation study (Figures 2 and 3). Results showed that ZINC000014811844 formed 10 pairs of hydrogen bonds with 2RCW, by the O2 of the compound with ARG217:HN of 2RCW, O2 of the compound with LEU216:HA of 2RCW, O31 of the compound with TYR235:HN of 2RCW, O24 of the compound with TYR246:HH of 2RCW, O26 of the compound with TYR246:HH of 2RCW, O31 of the compound with HIS201:HE1 of 2RCW, et al. (Table 5). Also, 3 pairs of π-related interactions were formed in the complex. ZINC000003938684 formed 5 pairs of π-related interactions with 2RCW, by 2 pairs of TYR228 of 2RCW with compound, 1 pair of TYR235 of 2RCW with compound, 1 pair of TYR228 of 2RCW with compound, and 1 pair of ARG227 of 2RCW with compound (Table 6). It also formed 17 hydrogen bonds with 2RCW. As for reference Olaparib, there are 2 hydrogen bonds with 2RCW and 6 π-related interactions with 2RCW.

**Table 4:** CDOCKER potential energy of compounds with CMET.

**Compounds**	**-CDOCKER potential energy (kcal/mol)**
ZINC000003938684	62.0446
ZINC000014811844	50.1851
Olaparib	49.0448

**Table 5:** Hydrogen bond interaction parameters for each compound with CMET.

**Receptor**	**Compound**	**Donor atom**	**Receptor atom**	**Distances (Å)**
2RCW	ZINC000003938684	ALA219:HN	ZINC000003938684:O19	3.09
		TYR228:HH	ZINC000003938684:O34	2.51
		TYR235:HN	ZINC000003938684:O27	1.88
		TYR246:HH	ZINC000003938684:O32	2.51
		ZINC000003938684:H61	ARG217:O	2.35
		ALA219:HA	ZINC000003938684:O19	3.03
		GLY227:HA2	ZINC000003938684:O36	2.48
		ZINC000003938684:H43	GLU327:OE2	2.85
		ZINC000003938684:H44	GLU327:OE1	2.57
		ZINC000003938684:H45	GLU327:OE1	2.8
		ZINC000003938684:H51	GLY233:O	2.35
		ZINC000003938684:H53	TYR228:OH	2.4
		ZINC000003938684:H54	ASP105:OD2	2.27
		ZINC000003938684:H60	ARG217:O	2.89
		ZINC000003938684:H60	GLY233:O	2.7
		ZINC000003938684:H71	GLU102:OE2	2.79
		ZINC000003938684:H73	GLU102:OE2	2.6
		TYR246:HH	ZINC000003938684	2.34
	ZINC000014811844	ARG217:HN	ZINC000014811844:O2	2.09
		TYR235:HN	ZINC000014811844:O31	2.65
		TYR246:HH	ZINC000014811844:O24	2.13
		TYR246:HH	ZINC000014811844:O26	2.18
		ZINC000014811844:H47	GLU102:OE2	2.12
		HIS201:HE1	ZINC000014811844:O31	2.72
		LEU216:HA	ZINC000014811844:O2	2.37
		ZINC000014811844:H42	GLY227:O	2.24
		ZINC000014811844:H48	GLY227:O	2.7
	Olaparib	Olaparib:H36	GLU102:OE2	2.27
		HIS248:HE1	Olaparib:O11	2.33

**Table 6:** π-Related interaction parameters for each compound with CMET.

**Compound**	**Donor atom**	**Receptor atom**	**Distances (Å)**
ZINC000003938684	TYR228	ZINC000003938684	3.94
	TYR246	ZINC000003938684	5.24
	ZINC000003938684:C21	ARG217	4.48
	TYR228	ZINC000003938684	5.47
	TYR235	ZINC000003938684	4.92
ZINC000014811844	TYR235	ZINC000014811844	5.19
	TYR235	ZINC000014811844	4.5
	HIS201	ZINC000014811844	5.63
Olaparib	ALA237	Molecule	4.01
	Olaparib	LYS242	4.47
	TYR235	Olaparib	4.71
	TYR246	Olaparib	4.44
	TYR246	Olaparib	5.04
	Olaparib	ARG204	5.45

### Molecular dynamics simulation

**Figure 4:**
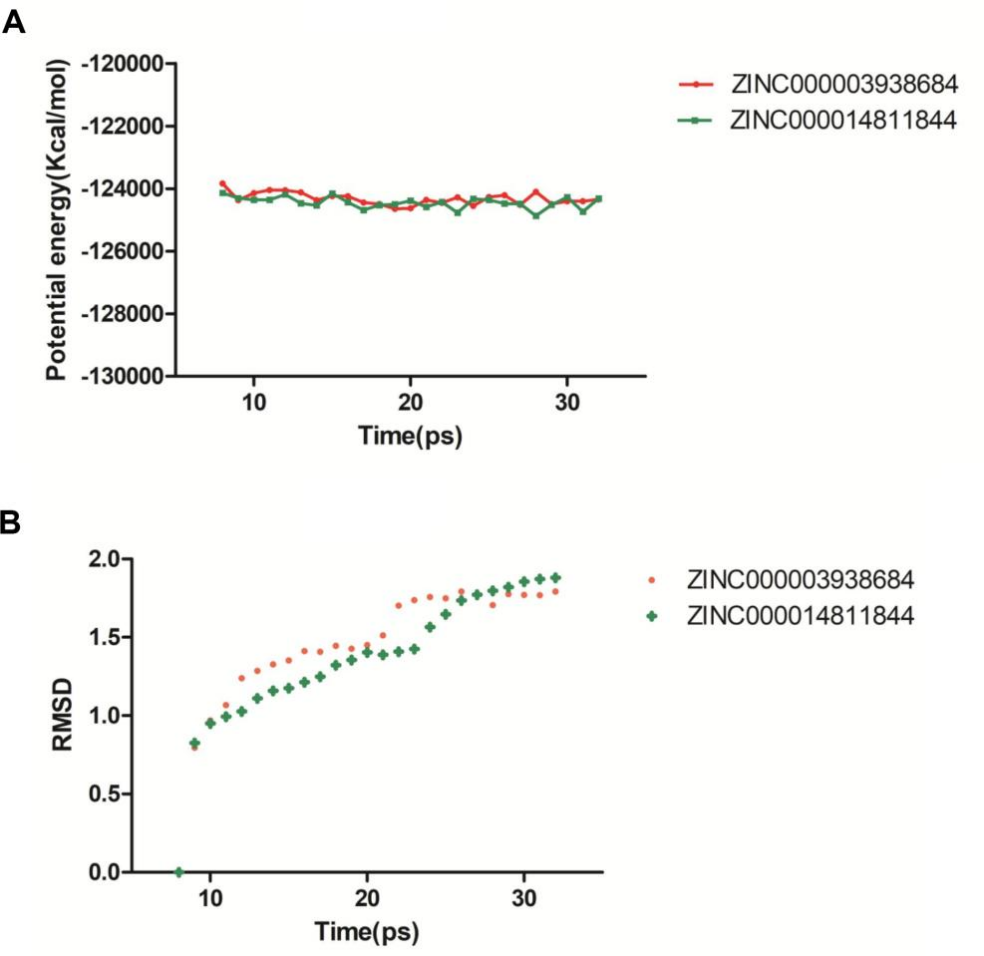
**Results of molecular dynamics simulation of compounds ZINC000003938684 and ZINC000014811844.** (**A**) Potential energy. (**B**) Average backbone root-mean-square deviation. RMSD: root-mean-square deviation.

For the sake of estimating the stabilities of the ligand-2RCW complexes in the natural environmental circumstances, a molecular dynamics simulation module was established. The molecular docking experiment was used to get the original conformations through the CDOCKER module. RMSD curves and potential energy chart of each complex were shown in Figure 4. After 30 ps, the trajectories of each complex reached equilibrium. With time going by, RMSD and potential energy of these complexes got stabilized gradually. Through molecular dynamics simulations, the hydrogen bond and p-dependent interactions between the compound and 2RCW were validated that they contribute to the stability of these complexes. To sum up, ZINC000003938684 and ZINC000014811844 could interact with 2RCW, and the complexes were stable in the natural environment which affected 2RCW.

## DISCUSSION

Glioblastoma (GBM) is the primary brain tumor with the highest incidence in the skull, among which glioblastoma has a very high degree of malignancy. Even after radiotherapy and chemotherapy, the median survival of patients is very short [4]. Protein PARP is one of the nuclear enzyme and plays a catalytic role in ribosylation of ADP. DNA in cancer cells leads to DNA damage under the action of therapeutic factors, such as radiotherapy and alkylating drugs, while PARP, as an intracellular DNA repair enzyme, can repair mutant damage in DNA, thus making the tumor resistant to these treatments [7]. Therefore, the key to inhibit tumor growth is to find an inhibitor of PARP to limit its activity, so as to resist tumor growth.

In recent years, the combination of PARP and other treatments that could lead to DNA damage in cancer cells (such as radiotherapy and chemotherapy) is a hot research field, which could enhance the efficacy of these treatments by weakening the ability to repair DNA damage in cancer cells [15]. At present, there are more than a dozen PARP inhibitors in preclinical or clinical research stage used as single-drug therapy or in combination with other targeted drugs or chemotherapy drugs to treat tumors. However, there are significant therapeutic limitations of the current inhibitors. PARP-2 is involved in the repair of DNA single-strand breaks, but its contribution to the total cellular level of PARP activity induced by DNA damage is very small (5-10%). These PARP inhibitors not only inhibit PARP1 but also inhibit the activity of PARP2 in varying degrees, resulting in side effects such as chronic anemia [16]. Actually, PARP2 plays important role in cancer development. According to research, PARP2 inhibitors can not only inhibite the repair of tumor cell DNA damage and promote tumor cell apoptosis as a single agent, but also enhance the efficacy of radiotherapy and chemotherapy with alkylating agents and platinum drugs [17]. Both PARP1 and PARP2 are involved in DNA damage response pathways and function as sensors of DNA breaks, including temporary single-strand breaks formed during DNA repair. Consistently, with a role in DNA repair, both PARP1 and PARP2 activation requires its binding to a damaged DNA site, which initiates PAR synthesis. PARP2 interacts with long DNA substrates containing a single damage site and representing intermediates of the short-patch base excision repair (BER) pathway. The functions of PARP1 and PARP2 overlap in BER after a site cleavage and PARP2 play a role in regulation of PARP1 activity [18]. Besides, the inhibitor target PARP2 also been verified effective to treat cancer such as breast cancer, ovarian cancer, hepatocellular carcinoma, cervical cancer [18–21]. So, though PARP inhibitors may result side effects, it also can make cancer cured by targeting PARP2.

Therefore, there is an urgent need to screen more compounds targeting PARP for clinical applications. In this research, Olaparib was selected as a reference drug in this study. Olaparib is the first FDA-approved PARP1/2 inhibitor for the treatment of ovarian cancer patients with BRCA gene deficiency [10].

In this study, LibDock, ADME, TOPKAT, CDOCKER and Molecular Dynamics Simulation, five sections of Discovery Studio were used for virtual screening and analysis. As a result, 17931 biogenic-for sale-named ligands were screened from the ZINC15 database for virtual screening. Compared with other compounds, compounds with a high LibDock score showed better energy optimization and a stable conformation. After the calculation of modules, 7894 compounds were found to be eligible to bind stably with 2RCW than Olaparib. The top 20 compounds were selected and pooled for further study based on the LibDock score.

ADME and toxicity predictions of the selected compounds were used to evaluate the pharmacologic properties of these compounds. Outcomes illustrated that ZINC000003938684 and ZINC000014811844 were regarded as safe drug candidates and chosen for the following study, since they had a good solubility level in water together with a good absorption level. Additionally, they didn’t have hepatotoxicity and they were non-inhibitors of cytochrome P450 2D6 (CYP2D6). Besides, these two compounds were also found to have less mutagenicity, rodent carcinogenicity and developmental toxicity potential compared with other compounds. Therefore, ZINC000003938684 and ZINC000014811844 were regarded as safe drug candidates. For another, the remaining drugs still had a possible function in drug development despite their possessed toxicities or negative effects. Given all the results above, ZINC000003938684 and ZINC000014811844 were selected as ideal lead compounds and further analysis was performed.

The bonding mechanism and chemical bonds of the selected candidate compounds were also researched. CDOCKER module computation illustrated that CDOCKER interaction energy of ZINC000003938684 and ZINC000014811844 was lower than the reference ligand Olaparib (-49.0448 kcal/mol), which could indicate that these two compounds had a higher binding affinity with 2RCW than Olaparib.

Finally, their stabilities in the natural environment were investigated by molecular dynamics simulation. Calculation of RMSD and potential energy of these ligand-2RCW complexes demonstrated the trajectories of complexes reached equilibrium after 30 ps. With time going by, RMSD and potential energy of these complexes got stabilized gradually, which showed ZINC000003938684 and ZINC000014811844 could interact with 2RCW and the complexes were stable in the natural environment. On account of the results, these 2 compounds could be used for drug development and refinement.

This study elucidated that the most important step in current drug designation was to screen ideal lead compounds. In this study, a battery of computer-aided virtual techniques was used to identify possible inhibitors of PARP. LibDock is applied for structure-based screening followed by ADME (absorption, distribution, metabolic, excretion) and toxicity prediction.

Molecular docking was conducted to confirm the binding affinity mechanism between the ligand and 2RCW. Molecular dynamics simulations were used to assess the stability of ligand-2RCW complexes. The results showed these 2 compounds might have a potential effect on glioblastoma. But it is all known that no single drug could be directly marketed without thousands of refinement and improvement. Therefore, the refinement and improvement of them are of great significance in the following research.

Discovery Studio is a professional, effective software for researchers to explore the optimal drug candidates in huge medication markets, the availability of this high-precision measurement had been verified in our previous study [22]. Though this study was well-designed and precise measurements have been conducted, it still has some shortcomings. Further experiments, for instance, MTT assay, flow cytometry, western blot and animal testing, need to be performed to further confirm our results, as well as discovery how and through which signaling pathway dose the inhibitor work. and more indicators, such as half-maximal inhibitory concentration, half-maximal effective concentration, LD50 and ED50 should be assessed in the future in order to make a comprehensive assessment to our inhibitors.

## CONCLUSIONS

This study conducted a battery of computer-aided structural and chemistry techniques (including virtual screening, ADME prediction, toxicity prediction, molecule docking simulation as well as dynamics simulation) to screen and identify the ideal lead compounds from huge number of natural drugs with functions to possibly inhibit PARP. After elaborate design and precise calculation, two compounds, ZINC000003938684 and ZINC000014811844, were selected as safe drug candidates, along with highly affinity to PARP, capability of competitive inhibition of PARP, as well as less developmental toxicity potential. Consequently, they played an important role in PARP inhibitor development. Besides, a list of drug candidates with pharmacologic properties was provided, which could make a great contribution to PARP or other proteins in medication design and improvement.

## MATERIALS AND METHODS

### Discovery studio software and ligand libraries

Discovery Studio is a suite of software to simulate small and large molecule systems, which is designed to screen, design and modify potential drugs through structural chemistry and structural biology calculations, thereby identifying and refining a wide range of lead compounds and candidate drug approaches [23]. The LibDock and ADME (absorption, distribution, metabolism, excretion) modules of Discovery Studio 4.5 software (DS4.5, Accelrys, Inc.) are applied in virtual screening. CDOCKER is used for docking research. Natural Products (NP) database in the ZINC15 database was used to screen PARP inhibitors as a selection. The Irwin and Shoichet laboratories, which is in the department of pharmaceutical chemistry at the University of California, San Francisco (UCSF), providing the ZINC database as a free commercial compound database.

### Use LibDock for structure-based virtual filtering

**Figure 5:**
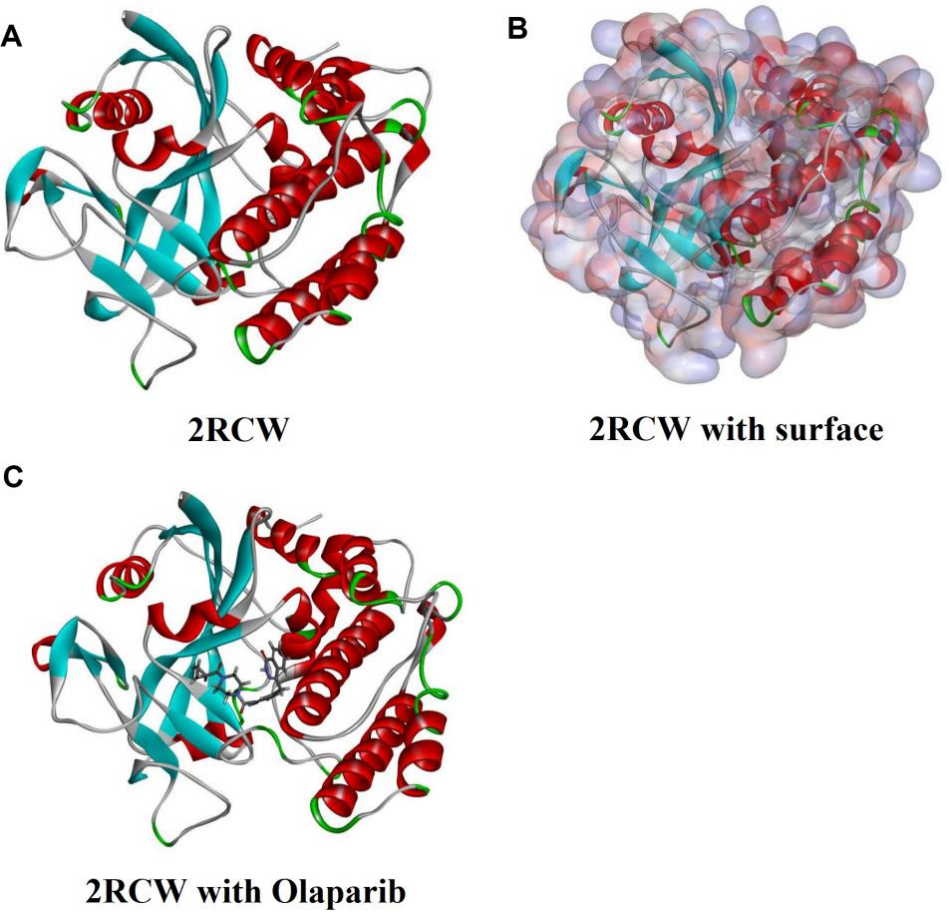
**Molecular structure of 2RCW (PARP complexed with A620223).** (**A**) Initial molecular structure. (**B**) Surface of binding area were added. Blue represents positive charge and red represents negative charge. (**C**) Molecular structure of Olaparib combined in binding area.

The ligand-binding pocket region of PARP was selected to identify new compounds that might inhibit PARP as the binding site. Additionally, this region is called the catalytic domain [24]. Virtual filtering is performed using the LibDock module of Discovery Studio 4.5 [25]. LibDock is a rigid docking program, which uses grids placed at binding sites and polar and non-polar probes to calculate protein hotspots. To form favorable interactions, the hotspots are furtherly used to align ligands, as well as the Smart Minimiser algorithm and CHARMm force field (Cambridge, Massachusetts, USA) for ligand minimization. All ligand positions were ranked by ligand scores after minimization. The 2.45Å crystal structure of PARP (Protein Data Bank identifier: 2RCW) and Olaparib (Protein Data Bank identifier: ZINC40430143) was downloaded from the Protein data bank (PDB) and imported into LibDock's work environment. The chemical structure of PARP is shown in Figure 5. Proteins are made by removing crystalline water and other heteroatoms and then adding hydrogen, protonation, ionization, and energy minimization. The CHARMm force field and Smart Minimiser algorithm were used to energy minimization [26]. With a root mean square(RSM) gradient tolerance of 12.277, 2000 steps were performed in the minimization with an, which resulted in an RMS gradient of 0.09778. To define binding sites the prepared proteins were used, the Olaparib binding site was selected as the active site for docking. By using LibDock, all prepared ligands were docked at defined active sites for virtual screening. According to the LibDock score, all docking positions are sorted and grouped by compound name.

### ADME (Absorption, Distribution, Metabolism, and Excretion) and toxicity prediction

The ADME module of Discovery Studio 4.5 is used to calculate the absorption, distribution, metabolism, and excretion of selected compounds, also used the DS4.5 TOPKAT (toxicity prediction by Computer assistive technology) module to calculate all potential compounds toxicity and other properties, including its water-soluble, blood-brain barrier (BBB) permeability, cytochrome P4502D6 (CYP2D6), liver toxicity, human intestinal absorption, plasma protein (PPB) levels, rodent carcinogenicity, ames respectively and developmental toxicity potential. These pharmacological properties should be taken into full consideration when selecting PARP drug candidates.

### Molecule docking and pharmacophore prediction

The CDOCKER module of Discovery Studio 4.5 was applied for molecular docking research. CDOCKER can produce high-precision docking results as a molecular docking method based on the CHARMm field. While allowing the ligand to bend during docking the receptor remains rigid. For each complex posture, the CHARMm energy (interaction energy plus ligand strain) and interaction energy indicated the ligand-binding affinity. From the protein database, the crystal structure of PARP can be obtained. During rigid and semi-flexible docking processes, crystallized water molecules were generally removed for fixed water molecules that may affect the formation of receptor-ligand complexes [27, 28]. Next, remove the water molecules and add the hydrogen atoms to the proteins. The initial compound, Olaparib, was extracted from the binding site and then realigned into the crystalline structure of PARP to demonstrate the reliability of the combination pattern. The force field of CHARMm36 was applied to the receptors and ligands. The definition of the binding site sphere of PARP was that of the region within a radius of 16 Å from the geometric center of mass of the ligand Olaparib. The ligand was combined with the residues in the binding spot during the docking. When it was ready to identify the hit structure, and docking it into the PARP binding pocket, the CDOCKER process was performed [29, 30]. Based on CDOCKER interaction, different postures of each test molecule can be analyzed.

### Molecular dynamics simulation

The best binding conformations of each compounds-2RCW complex were chosen for molecular dynamics simulation. an orthorhombic box was built for the ligand-receptor complex was put into an orthorhombic box and solvated with an explicit periodic boundary solvation water model. Solidum (ionic strength of 0.145) chloride was poured into the system for the sake of simulating the physiological environment. Then the CHARMM force field and energy minimization were prepared for the system (500 steps of steepest descent and 500 steps of conjugated gradient), with a result showing that the final root means square gradient of 0.227. The system was slowly driven from an initial temperature (296K) to the target temperature(320K) in 2 ps, and equilibration simulations were performed for 5 ps. Molecular dynamics simulation (production module) was run for 25 ps and the time step was 1 fs. The simulation was run with the normal pressure and temperature system (300K) during the process. Long- range electrostatics were calculated by the particle mesh Ewald algorithm, and all bonds involving hydrogen were fixed by the linear constraint solver algorithm. Select initial complex setting as a reference, Discovery Studio 4.5 analysis trajectory protocol was used for a trajectory determined for RMSD, potential energy, and structural characteristics.

### Availability of data and materials

The datasets used and/or analyzed during the current study are available from the corresponding author on reasonable request.
